# http://www.euro-wabb.org: an EU Register for Alstrom, Bardet Biedl and other rare syndromes

**DOI:** 10.1186/2046-2530-1-S1-P2

**Published:** 2012-11-16

**Authors:** T Barrett, A Farmer, S Aymé, P Maffei, S McCafferty, W Mlynarski, V Nunes, V Paquis, K Parkinson, J Rohayem, R Sinnott, V Tillmann, L Tranebjaerg

**Affiliations:** 1 University of Birmingham, UK; 2 Birmingham Children's Hospital, UK; 3 INSERM, France; 4 University of Padova, Italy; 5 University of Glasgow, UK; 6 University of Lodz, Poland; 7 IDIBELL, Spain; 8 Centre Nationale de la Recherche Scientifique, France; 9 Alstrom Syndrome UK; 10 University of Dresden, Germany; 11 University of Melbourne, Australia; 12 University of Tartu, Estonia; 13 University of Copenhagen, Denmark

## 

Alstrom syndrome (infancy onset obesity, cardiomyopathy, retinal dystrophy and renal complications) and Bardet Biedl syndrome (polydactyly, infancy onset obesity, retinal dystrophy and learning difficulties) are uncommon (less than 1:100,000), linked by obesity, vision loss and deafness, frequently develop diabetes mellitus by adulthood, and share ciliopathy as the underlying pathology. Delayed diagnosis is common; treatable complications are often missed; and access to molecular genetic testing is unequal between European citizens. There are as yet no orphan drug treatments available, and no access to well characterized cohorts of patients to undertake research. We aimed to establish a European Registry to address these issues. We agreed a common dataset of clinical, investigation and molecular diagnostic data to distinguish between these and other rare syndromes. We wrote an ethics submission template for national approvals, to include consent to link national and international registries. We designed a web based registry with built in security for data confidentiality, anonymised data collection, and facility for patients to self register. Finally we designed a website for dissemination of information to health professionals and families. The core dataset includes 44 data fields which define and separate the syndromes; the extended dataset comprises 370 fields of detailed phenotyping information. We currently have ethics approval in 6 EU states, and the first 40 patients consented, mainly from Italy and UK. This EU Registry will aid the development of national management guidelines and education material for health professionals; improve patient services, raise awareness, and allow recruitment into multi-national clinical trials.

http://www.euro-wabb.org

